# The Microbiome and Occurrence of Methanotrophy in Carnivorous Sponges

**DOI:** 10.3389/fmicb.2016.01781

**Published:** 2016-11-09

**Authors:** Jon T. Hestetun, Håkon Dahle, Steffen L. Jørgensen, Bernt R. Olsen, Hans T. Rapp

**Affiliations:** ^1^Marine Biodiversity Group, Department of Biology, University of BergenBergen, Norway; ^2^Centre for Geobiology, University of BergenBergen, Norway; ^3^Uni Research Environment, Uni Research ASBergen, Norway

**Keywords:** Porifera, Cladorhizidae, Barbados, Arctic Mid-Ocean Ridge, hydrothermal vent, methane seep, isotope, *Cladorhiza methanophila*

## Abstract

As shown by recent studies, filter-feeding sponges are known to host a wide variety of microorganisms. However, the microbial community of the non-filtering carnivorous sponges (Porifera, Cladorhizidae) has been the subject of less scrutiny. Here, we present the results from a comparative study of the methanotrophic carnivorous sponge *Cladorhiza methanophila* from a mud volcano-rich area at the Barbados Accretionary Prism, and five carnivorous species from the Jan Mayen Vent Field (JMVF) at the Arctic Mid-Ocean Ridge. Results from 16S rRNA microbiome data indicate the presence of a diverse assemblage of associated microorganisms in carnivorous sponges mainly from the Gamma- and Alphaproteobacteria, Flavobacteriaceae, and Thaumarchaeota. While the abundance of particular groups varied throughout the dataset, we found interesting similarities to previous microbiome results from non-carnivorous deep sea sponges, suggesting that the carnivorous sponges share characteristics of a previously hypothesized putative deep-sea sponge microbial community. Chemolithoautotrophic symbiosis was confirmed for *C. methanophila* through a microbial community with a high abundance of Methylococcales and very light isotopic δ^13^C and δ^15^N ratios (-60 to -66‰/3.5 to 5.2‰) compared to the other cladorhizid species (-22 to -24‰/8.5 to 10.5‰). We provide evidence for the presence of putative sulfur-oxidizing Gammaproteobacteria in the arctic cladorhizids; however, δ^13^C and δ^15^N signatures did not provide evidence for significant chemoautotrophic symbiosis in this case, and the slightly higher abundance of cladorhizids at the JMVF site compared to the nearby deep sea likely stem from an increased abundance of prey rather than a more direct vent association. The phylogenetic position of *C. methanophila* in relation to other carnivorous sponges was established using a three-gene phylogenetic analysis, and it was found to be closely related to other non-methanotrophic *Cladorhiza* species with a similar morphology included in the dataset, suggesting a recent origin for methanotrophy in this species. *C. methanophila* remains the only known carnivorous sponge with a strong, chemolithoautotrophic symbiont association, and methanotrophic symbiosis does not seem to be a widespread property within the Cladorhizidae.

## Introduction

Sponges (Porifera) are among the earliest diverging animals and comprise one of the major animal phyla, with over 8,700 species currently described (van Soest et al., 2016). They have been shown to often live in close association with abundant and highly diverse microbial communities, which can account for up to 35–40% of sponge biomass in some cases (Taylor et al., 2007; Hentschel et al., 2012). Several studies, in recent years greatly facilitated by the use of next generation sequencing techniques, have identified an extraordinary diversity of microorganisms in sponges from all three domains of life. More than 30 bacterial phyla as well as archaeal and single-celled eukaryotes have been reported, including a candidate phylum, Poribacteria, which is known almost exclusively from sponges (Fieseler et al., 2004; Lafi et al., 2009; Hentschel et al., 2012; Thomas et al., 2016). Within a single sponge, one may find hundreds to thousands of unique microbial operational taxonomic units (OTUs), some of which are common in marine environments, but many of which tend to be specific to a single host species or group of sponges (Hentschel et al., 2012; Thomas et al., 2016).

The vast majority of sponges obtain nutrients by filtering water through an aquiferous system, and take up particles through choanocyte cells into the interior mesohyl of the sponge where digestion occurs. The mesohyl, an extracellular matrix making up the bulk of the sponge, is populated by motile sponge cells, and provides a habitat for the associated microbiome of the sponge (Bergquist, 1978; Taylor et al., 2007). Microorganisms benefit from an increased access to nutrients, while in turn providing a variety of functions relating to the metabolism of the host sponge such as nitrogen cycling, carbon fixation, and the production of a vast array of secondary metabolites (e.g., Taylor et al., 2007; Hoffmann et al., 2009). Thus, a complete understanding of sponge function is incomplete without taking into account its microbial community, and a sponge could most accurately be considered as the sum of its host cells and associated microbiome.

Carnivorous sponges, comprising family Cladorhizidae Dendy, 1922 (Demospongiae, Poecilosclerida), are the only sponges known to have a partly or completely reduced aquiferous system. These sponges are instead able to capture and engulf prey such as small crustaceans and other planktonic organisms. Cladorhizids are usually erect, with a stalked, pennate, or branching morphology. Prey items become stuck to the adhesive surface and filamentous appendages of the sponge and motile cells migrate to and then envelop the prey, in effect creating a temporary digestive cavity around it (e.g., Vacelet and Boury-Esnault, 1995; Kübler and Barthel, 1999; Vacelet and Duport, 2004; Vacelet, 2007). Currently, around 150 species are recognized, belonging to the nine genera *Abyssocladia* Lévi, 1964 (around 20 spp.), *Asbestopluma* Topsent, 1901 (around 30 spp.), *Cercicladia* Ríos et al., 2011 (1 sp.), *Chondrocladia* Thomson, 1873 (around 35 spp.), *Cladorhiza* Sars, 1872 (around 35 spp.), *Euchelipluma* Topsent, 1909 (around 5 spp.), *Koltunicladia* Hestetun et al., 2016b (1 sp.), *Lollipocladia* Vacelet, 2008 (1 sp.), and *Lycopodina* Lundbeck, 1905 (around 25 spp.) (Hestetun et al., 2016b; van Soest et al., 2016).

A remnant aquiferous system is found in genus *Chondrocladia*, where it is used to inflate spherical swellings for use in prey capture and reproductive purposes rather than filter-feeding (Kübler and Barthel, 1999; Lee et al., 2012), and other carnivorous sponges completely lack this structure. Transmission electron microscopy (TEM) images indicate at least three bacterial morphotypes in the species *Cladorhiza methanophila* Vacelet and Boury-Esnault, 2002 from a mud volcano at the base of the Barbados Accretionary Prism (BAP) (Vacelet et al., 1995; Vacelet and Boury-Esnault, 2002), and reported TEM observations from the Pacific species *Lycopodina occidentalis* (Lambe, 1893) show that bacteriocytes are abundant in the mesohyl, especially in the filaments, in species without known chemoautotrophic symbionts as well (Riesgo et al., 2007). However, the only known microbiome data currently available for carnivorous sponges is from Dupont et al. (2013, 2014), who characterized the microbial community of *L. hypogea* Vacelet and Boury-Esnault, 1996 from a Mediterranean cave.

Carnivorous sponges are also of special interest due to the fact that methanotrophic bacteria have been reported in *C. methanophila* (Vacelet et al., 1995; Vacelet and Boury-Esnault, 2002). In contrast to more well-known chemoautotrophic symbioses in groups such as polychaetes, bivalves or crustaceans (e.g., Felbeck, 1981; Childress et al., 1986; Petersen and Dubilier, 2009), this is one of only a handful known or suspected vent or seep related chemoautotrophic symbioses within Porifera (Arellano et al., 2013).

Here, we present the results of a comparative study of the microbiomes of 11 specimens representing five cladorhizid species from the Jan Mayen Vent Field (JMVF) on the Arctic Mid-Ocean Ridge including the genera *Asbestopluma*, *Cladorhiza*, and *Lycopodina*, and four specimens representing the species *C. methanophila* from the BAP, which is known to harbor methanotrophic symbionts. Our aims were to (1) provide additional data on the microbiome composition of carnivorous sponges and compare this to previous sponge microbiome studies, and (2) to compare the microbiome of a known methanotrophic carnivorous sponge to that of other, related carnivorous species and explore the extent of methanotrophy in the sampled species.

In order to reach these aims, microbial community structures within sponge specimens were analyzed by sequencing of 16S rRNA gene amplicons with the Ion Torrent sequencing technology, using primers universal for Bacteria and Archaea, with subsequent amplicon taxonomic assignment and ecological analysis. The ratio of heavy isotopes of e.g., carbon and nitrogen is a common tool for studying food sources and trophic interactions. Very light isotopic ratios indicate organisms with prokaryote symbiosis (Childress et al., 1986; Fry, 2007), and the microbiome data was supplemented with δ^13^C and δ^15^N stable isotope signatures in order to further investigate methanotrophic or other chemoautotrophic symbiosis in the sample material. Finally, the systematic position of *C. methanophila* was determined relative to the other carnivorous species based on a phylogenetic analysis also including other available cladorhizid sequences from GenBank.

## Materials and Methods

### Study Area

**FIGURE 1:**
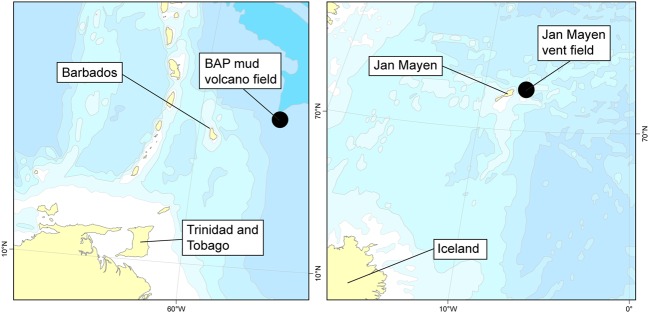
**Collection of samples included in this study.** Left: Diapiric field at the base of the Barbados Accretionary Field (BAP). Right: Jan Mayen Vent Field on the southwestern Mohns Ridge, part of the Arctic Mid-Ocean Ridge.

**FIGURE 2:**
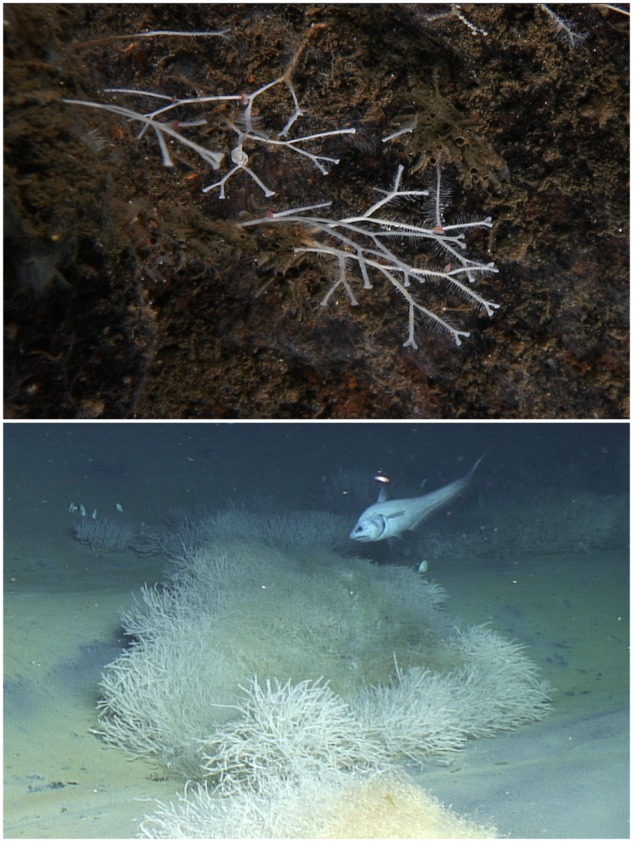
***In-situ* images of *Asbestopluma furcata* (top) from the Troll Wall vent site (JMVF) and *Cladorhiza methanophila* (bottom) from the Atalante mud volcano (BAP).** The BAP photo is courtesy of B. Ball, taken by ROV *Jason* operated by the Woods Hole Oceanographic Institution.

Specimens were collected from two locations: the Mount Manon, Atalante, and Volcano A mud volcanoes at the base of the BAP (~4700–4900 m) at 13°50′N (Le Pichon et al., 1990; Olu et al., 1997) (*C. methanophila*), and the JMVF on the ultraslow-spreading Arctic Mid-Ocean Ridges at the Jan Mayen fracture zone at approximately 71°15′N and 005°45′W (Pedersen et al., 2010; Schander et al., 2010) (all other cladorhizids) (**Figure 1**). The mud volcano field at the base of the BAP features massive methane emission from diapiric structures with associated seep fauna mostly belonging to bivalves with chemosynthetic symbionts in additions to large bush-like aggregations of *C. methanophila* (Olu et al., 1997). Samples from the JMVF were taken mainly from the Troll Wall site (~71.25°N), a part of the rift valley close to Jan Mayen, with an additional specimen from an area of ultradiffuse venting in the rift valley characterized by biofilms dominated by *Mariprofundus ferrooxydans* (Pedersen et al., 2010) (**Figure 2**). Cladorhizids are common in the vicinity of venting sites at the JMVF, but despite the presence of free-living chemoautotrophic microorganisms at these sites (e.g., Lanzén et al., 2011) the lack of any large aggregations of biomass like those found at the BAP, could indicate that cladorhizids here benefit more indirectly from the increased prey availability at these sites (Vacelet, 2006) rather than directly deriving nutrition from chemoautotrophic symbionts.

### Specimen Collection

Specimens used for microbiome sequencing were collected primarily using the *Bathysaurus* ROV during the University of Bergen Centre for Geobiology (CGB) R/V “G.O. Sars” 2011, 2012, and 2014 cruises to the JMVF, and with the *Jason* ROV during the 2012 R/V “Atlantis” 21-02 cruise to the base of the BAP. Isotope sampling included additional specimens from CGB “G.O. Sars” 2008–2014 cruises. The majority of specimens were either preserved by snap-freezing samples in liquid nitrogen (LN2), or in RNAlater. RNAlater specimens were as a rule kept at normal freezer temperatures (-20°C) during storage. We were also interested in seeing if a couple of ethanol preserved samples were viable for microbiome sequencing, and for some specimens, we ran parallels from part of the specimen fixed in 96% ethanol, and for two specimens, we sequenced from ethanol preserved material only. A list of cladorhizid specimens used for microbiome sequencing and host phylogenetic analysis is given in **Table 1**. Specimens used for isotope analysis are listed together with the results of the analysis in **Table 2**.

**Table 1:** List of samples analyzed in the current study.

Species	Cruise	Specimen ID	Locality	Sample ID
*Asbestopluma furcata*	CGB2014	GS14-ROV11-2	Troll Wall (JMVF)	Afur1
*A. furcata*	CGB2014	GS14-ROV11-3	Troll Wall (JMVF)	Afur2
*Cladorhiza abyssicola*	CGB2012	GS12-ECO2-B-ROV08-01	Troll Wall (JMVF)	Caby1
*C. corticocancellata*	CGB2011	GS11-ROV06-02	JMVF rift valley	Ccor1
*C. gelida*	CGB2014	GS14-AGT06-16	East of JMVF	Cgel1
*C. gelida*	CGB2014	GS14-AGT08-12	East of JMVF	Cgel2
*C. methanophila^∗^*	Atlantis 21-02	J2-633-5	BAP	Cmet1
*C. methanophila*	Atlantis 21-02	J2-633-4	BAP	Cmet2
*C. methanophila*	Atlantis 21-02	J2-634-10	BAP	Cmet3
*C. methanophila^∗^*	Atlantis 21-02	J2-636-6	BAP	Cmet4
*Lycopodina cupressiformis*	CGB2014	GS14-ROV11-4	Troll Wall (JMVF)	Lcup1
Water sample	CGB2014	NA	Troll Wall (JMVF)	Wat1

*Individual specimens are referred to by sample ID throughout the text. ^∗^These specimens were also used in the phylogenetic analysis in this study*.

**Table 2:** Stable isotope values of carbon (δ^13^C) and nitrogen (δ^15^N) of cladorhizid specimens.

Species	Specimen	Locality		d^13^C (‰)	d^15^N (‰)
*A. furcata*	GS11-ROV07-02	JMVF	Troll Wall	-22.087	10.630
*A. furcata*	GS12-ROV04-03	JMVF	Troll Wall	-25.960	10.953
*A. furcata*	GS12-ROV04-15	JMVF	Troll Wall	-21.489	9.944
*A. furcata*	GS14-ROV11-02	JMVF	Troll Wall	-25.796	9.384
*C. abyssicola*	GS12-ROV08-01	JMVF	Troll Wall	-23.417	8.573
*C. corticocancellata*	GS11-ROV06-02	JMVF	G. Garden	-22.671	8.413
*L. cupressiformis*	GS11-ROV07-13	JMVF	Troll Wall	-22.647	11.834
*L. cupressiformis*	GS11-ROV07-11	JMVF	Troll Wall	-20.821	9.362
*L. cupressiformis*	GS12-ROV04-07	JMVF	Troll Wall	-23.001	12.351
*L. cupressiformis*	GS12-ROV14-13	JMVF	Troll Wall	-20.970	9.818
*L. cupressiformis*	GS14-ROV11-04	JMVF	Troll Wall	-24.295	13.295
*C. methanophila*	J2-633-5	BAP	Manon	-62.660	4.185
*C. methanophila*	J2-633-4	BAP	Manon	-64.601	3.513
*C. methanophila*	J2-634-10	BAP	Volcano A	-65.951	5.225
*C. methanophila*	J2-636-3	BAP	Atalante East	-60.498	3.619

*Specimens were collected from the JMVF except C. methanophila specimens, which were collected at the base of the BAP*.

### Stable Isotope Analysis

Stable isotope analyses (δ^13^C and δ^15^N) were conducted at the University of Bergen. Samples were dried at 80°C for 24 h in glass vials and grounded to powder using a glass pestle. Lipids were removed by adding 7% methanol in dichloromethane for 2 h to the dried sample and then removed using a glass Pasteur pipette and again dried for 24 h at 80°C. Inorganic carbon was removed by adding 0.5 M HCl to the samples. The time for the inorganic carbon to dissolve varied and the HCl was kept at minimum 5 min or until the reaction was finished. Acid waste was washed away by carefully adding and removing water to the sample until the pH reached 6–7. Finally the samples were dried and weighed in tin capsules and measured using a Delta V Plus isotope ratio mass spectrometer connected to a Flash EA 1112 elemental analyzer (Thermo Scientific). Isotope ratios are expressed in delta notation as ‰ difference in ^13^C/^12^C and ^15^N/^14^N isotope ratios compared to Pee Dee Belemnite (PDB) and air N2, respectively. Samples were calibrated to internationally acknowledged C and N isotope ratio standards.

### DNA Extraction

Specimen subsamples were examined and visible contaminants removed and rinsed several times using Milli-Q water in a sterile environment. Extraction of grinded tissue was done using either the E.Z.N.A. Mollusc DNA kit (Omega Bio-tek) or the Qiagen Blood and Tissue kit. A water sample from the JMVF was filtered through a 0.22 μm cellulose filter that was cut into small pieces before extraction. Spicules were removed from the extract after lysis before adding ethanol.

### *Cladorhiza methanophila* Sanger Sequencing

In order to establish the phylogenetic position of *C. methanophila*, partial ribosomal 28S rRNA, mitochondrial gene cytochrome c oxidase subunit I (COI) and nuclear gene asparagine-linked glycosylation protein 11 (ALG11) were sequenced to allow comparison with existing cladorhizid sequences in GenBank from Hestetun et al. (2016b), which already includes other cladorhizids part of our study. A maximum likelihood analysis using RAxML 8.0.20 (Stamatakis, 2014) was performed on a concatenated dataset with the resulting ML most likely tree pictured with rapid bootstrap values (BS) indicated. Amplification procedure and analysis are identical to that of Hestetun et al. (2016b), where they are described in further detail.

### Ion Torrent 16S Amplicon Sequencing

For 16S rDNA sequencing, we targeted the V4–V5 regions using the universal prokaryote primers 519F (5′-CAGCMGCCGCGGTAA-3′) (Øvreås et al., 1997) and 805R (5′-GACTACHVGGGTATCTAATCC-3′) (Herlemann et al., 2011). Ion Torrent libraries were constructed as described in detail in Jørgensen and Zhao (2016). In short: all DNA extracts were PCR amplified in triplicates and subsequently pooled and purified using QIAquick PCR purification kit (Qiagen). The purified DNA was used in a second PCR in order to attach barcodes and adaptor sequence. Resulting amplicons were purified using AMPure XP bead Purification Kit (Agencourt), following the manufacturer’s protocol, before all samples were pooled in equimolar concentrations (26 pmol). Prior to sequencing quantification was done using a Quantus Fluorometer. Amplicons were sequenced applying the Ion Torrent PGM Personal Genome Machine (PGM) platform technology (Life Technologies) with a 318 chip. The thermal PCR programs used were as follow: first round PCR: each reaction (20 μl) contained 10 μl 2x HotStarTaq^®^master mixture (Qiagen), 0.2 μl of each primer (100 μM stock), 2 μl template, and ddH_2_O. The PCR program was initiated with a hot start activation step for 15 min at 95°C followed by 27 cycles of 95°C for 30 s, 56°C for 30 s, and 72°C for 30 s. Second round PCR used same program but only seven cycles and contained in each reaction (25 μl) contained 12.5 μl 2x HotStarTaq^®^master mixture (Qiagen), 0.2 μl 806r-B-Key (100 μM stock), and 2 μl 519f MID primer (10 μM stock), with 5 μl of purified PCR products from first-round amplification as the template, according to the Ion Torrent protocol.

### OTU Clustering, Taxonomic Assignment, and Data Treatment

Filtering (truncate length 200, max expected errors 0.5), chimera detection, removal of singletons, and OTU clustering (97% identity) were done using the UPARSE pipeline with USEARCH 8.1.1861 (Edgar, 2013). Taxonomic assignment and phylogenetic tree construction was done with the Green Genes database v 13_8 using QIIME (Caporaso et al., 2010), and 16S rRNA amplicon data was submitted to the Sequence Read Archive as biosamples with accession numbers SAMN05722969 and SAMN05722971–SAMN05722981, under BioProject accession number PRJNA341449. Bray–Curtis pairwise dissimilarity index values, cluster and MDS analyses were calculated using R package vegan, and data were visualized using R packages ggplot2 and pheatmap. *C. methanophila* Sanger sequences for specimens J633-5 (Cmet1) and J636-3 (Cmet4) were uploaded to GenBank with accession numbers KX815331–KX815335.

## Results

### Stable Isotope Analysis

**FIGURE 3:**
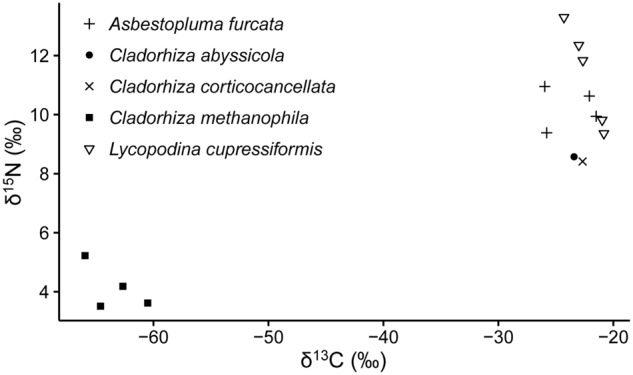
**Stable isotope values of carbon (δ^13^C) and nitrogen (δ^15^N) of a selection of cladorhizids collected at the Troll Wall vent field, and the BAP diapiric field**.

Stable isotope analysis of δ^13^C and δ^15^N was done on 15 samples representing five cladorhizid species (**Figure 3**; **Table 2**). The results show that all JMVF cladorhizids had isotope ratios within a quite narrow range of around -22 to -24‰ for δ^13^C and 8.5 to 10.5‰ for δ^15^N. The BAP *C. methanophila* specimens were found to have very light δ^13^C and δ^15^N ratios of around -60 to -66‰ for δ^13^C and 3.5 to 5.2‰ for δ^15^N, clearly separating it from the other cladorhizid specimens (Mann–Whitney rank-sum *p*-value = 0.001465).

### Host Phylogenetic Analysis

**FIGURE 4:**
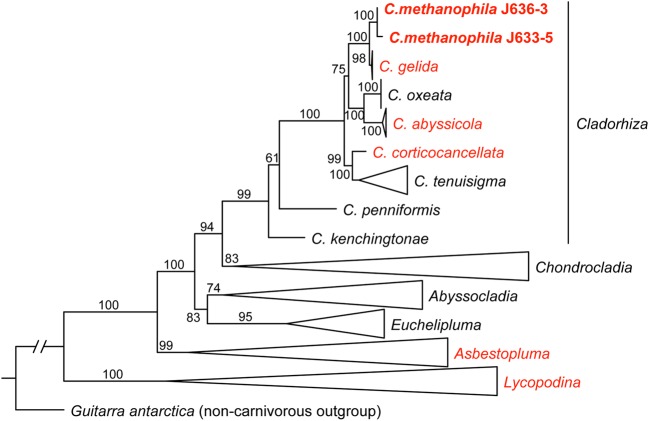
**A maximum likelihood (ML) analysis of the carnivorous sponge family Cladorhizidae based on a concatenated dataset of partial 28S rDNA, COI and ALG11 (2859 bp), showing the position of *C. methanophila* (bold).** Taxa in red include species subject to 16S rRNA amplicon sequencing in this study. ML bootstrap value are indicated for each branch. The tree is rooted using the non-carnivorous sponge *Guitarra antarctica* (Guitarridae). Genera except *Cladorhiza* are collapsed. Taxa except *C. methanophila* (*N* = 105) were retrieved from GenBank, and were originally published in Vargas et al. (2013); Riesgo et al. (2014) and Hestetun et al., 2016a,b.

The Cladorhizidae phylogenetic analysis recovered the two sequenced *C. methanophila* specimens as a close sister taxon to *C. gelida* with high support (BS = 100). *C. gelida* is a common *Cladorhiza* species with a wide biogeographical distribution and eurybathyal depth range, and is also included in the microbiome dataset here. Other North Atlantic arbuscular *Cladorhiza* species (*C. abyssicola* Sars, 1872*, C. oxeata* Lundbeck, 1905, *C. corticocancellata* Carter, 1876, and *C. tenuisigma* Lundbeck, 1905) were also recovered as close relatives within the genus (**Figure 4**).

### 16S rRNA Gene Sequencing Analyses

#### Run Statistics

We obtained 16S rRNA sequences from 20 samples representing 11 specimens and one water sample. Filtering removed 36.6% of initial unfiltered reads, and the total number of filtered reads was 2,067,945, with individual run ID reads ranging between 25,987 and 207,572. Total number of unique reads was 349,835. Filtered reads were clustered into 2,005 OTUs using a 97% identity threshold (**Table 3**). Multiple replicates from the same specimen were pooled together in subsequent analyses. In order to mitigate biases from variable sequencing depth, pooled samples were subsampled to a standard depth of 45,000 reads for all subsequent analyses (smallest pooled sample was ~49,000 reads).

**Table 3:** Dataset overview for 16S rRNA gene sequencing samples in this study.

Sample	Fixation	Locality	Run ID	Total reads	Filtered reads	% filtered	Unique reads	Singletons	Unique OTUs (97%)
***A. furcata***									
Afur1	LN2	JMVF	1–2	50,407	36,109	28.4	8,973	7,238	44
Afur1	LN2	JMVF	1–3	66,456	45,949	30.9	10,434	8,272	62
Afur2	LN2	JMVF	2–9	268,401	201,107	25.1	32,764	25,200	182
***C. abyssicola***									
Caby1	EtOH	JMVF	1–4	49,509	32,217	34.9	6,282	4,882	41
***C. corticocancellata***									
Ccor1	EtOH	JMVF	1–5	60,267	38,253	36.5	7,216	5,612	36
***C.gelida***									
Cgel1	RNAlater	JMVF	2–10	238,967	141,606	40.7	23,651	18,289	167
Cgel2	EtOH	JMVF	2–11	198,932	119,531	39.9	20,708	16,264	134
Cgel2	LN2	JMVF	2–12	259,974	142,850	45.1	25,129	19,819	183
Cgel2	LN2	JMVF	2–13	254,578	169,446	33.4	27,509	21,009	194
***C. methanophila***									
Cmet1	EtOH	BAP	2–1	188,380	124,501	33.9	37,418	30,916	184
Cmet1	RNAlater	BAP	2–2	119,442	77,081	35.5	25,064	20,905	134
Cmet1	RNAlater	BAP	2–3	112,783	74,217	34.2	25,152	20,907	118
Cmet2	EtOH	BAP	2–4	292,129	176,742	39.5	50,169	40,843	248
Cmet2	RNAlater	BAP	2–5	173,029	105,972	38.8	30,285	24,765	184
Cmet3	RNAlater	BAP	2–6	222,248	137,885	38.0	38,004	30,838	219
Cmet4	RNAlater	BAP	2–7	215,284	134,696	37.4	34,992	28,307	181
Cmet4	RNAlater	BAP	2–8	336,767	207,572	38.4	50,134	40,013	229
***L. cupressiformis***									
Lcup1	LN2	JMVF	1–1	53,611	34,971	34.8	10,778	8,854	89
**Water sample**									
Wat1	LN2	JMVF	1–6	43,103	25,987	39.7	14,366	12,097	322
Wat1	LN2	JMVF	1–7	69,105	41,253	40.3	19,893	16,456	428
**Aggregated**				3,273,372	2,067,945	36.3	349,835	267,890	2,005

*Multiple samples from the same specimen are indicated with specimen numbering and color. Run ID comprises run (1 or 2) and MID. Unique OTUs are with singletons and chimeras removed. Statistics for aggregated run lists unique reads, singletons and unique OTUs across the entire dataset. Samples from the same specimen were pooled together, and sequencing depth standardized to 45000 reads, for subsequent analyses*.

**FIGURE 5:**
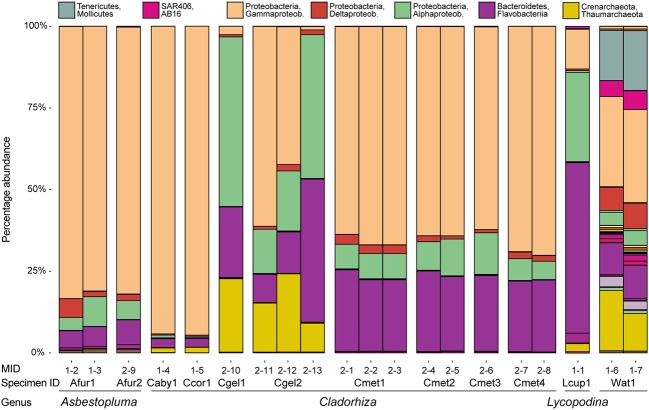
**Microbial abundance for individual samples (MIDs) at the class level for the purposes of assessing similarity between individual samples for the same species.** Also indicated is specimen ID and genus assignment. Samples from the same specimen were pooled in subsequent analyses.

Checking for any systematic bias in taxonomic assignments due to extraction kit, we did not see any consistent difference between samples extracted with the Qiagen kit (Afur1 1–3, Afur2, Caby1, Ccor1, Cgel1, Cgel2 2–13, Cmet4 2–8) and the E.Z.N.A. Mollusc kit (Afur1 1–2; Cgel2 2–11 and 2–12; all Cmet1, Cmet2, and Cmet3; Cmet4 2–7; Lcup1; Wat1) (**Figure 5**). Looking at the viability of EtOH as fixative (in this particular case, with comparatively fresh specimens kept in cold storage) for samples Caby1, Ccor1, Cgel2 2–11, Cmet1 2–1, and Cmet2 2–4, we found no difference for the Cmet samples, but samples Caby1, Ccor1, and Cgel2 showed a comparatively large abundance of Thiohalorhabdales (Gammaproteobacteria) compared to other samples (and replicates in the case of Cgel2) (**Figure 5**). While the variable abundance of Thiohalorhabdales is consistent with earlier results showing that prokaryote concentration is variable throughout the sponge (see section “Comparison with Other Sponge Microbiomes”), and could thus be attributable to sampling location as the Thiohalorhabdales abundance is also variable for the two non-EtOH replicates of Cgel2, it is also possible that the EtOH fixative has influenced relative abundance for these three samples, which has been taken into account in the relevant discussion.

#### Microbial Community Structure

**FIGURE 6:**
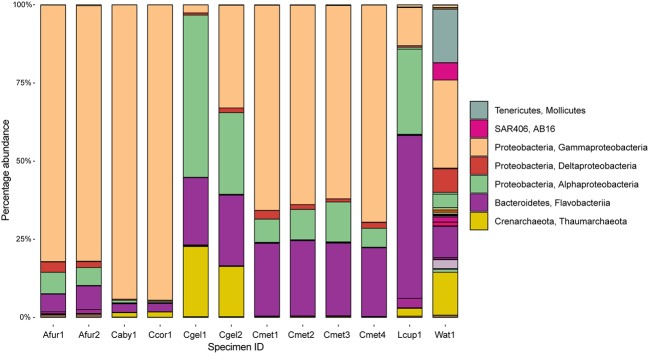
**Abundance at the class level across sampled cladorhizids and Troll Wall water sample.** Major classes are indicated in the legend.

**FIGURE 7:**
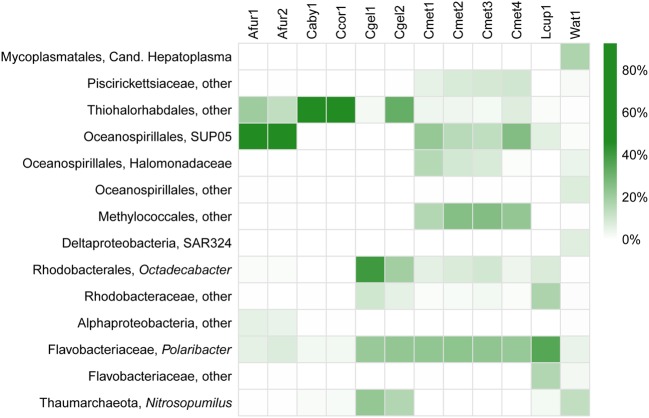
**Heatmap of relative abundance (%) of the most common prokaryote groups in the dataset at genus level**.

At the phylum level, 97% OTUs were classified into 41 bacterial and 2 archaeal phyla, with between 15 and 36 phyla for individual samples. The most common prokaryotes at class level are given in **Figure 6**. The 10 most common groups at genus level are given in table form in **Table 4**, and a heatmap showing overlap in identity between specimens at genus level is given in **Figure 7**.

**Table 4:** Abundance of the 10 most common prokaryotes over the whole dataset at the genus level.

	Total	Afur1	Afur2	Caby1	Ccor1	Cgel1	Cgel2	Cmet1	Cmet2	Cmet3	Cmet4	Lcup1	Wat1
Thiohalorhabdales, unknown, unknown	**22.5%**	**20.2%**	**12.8%**	**92.0%**	**92.6%**	2.2%	**32.3%**	3.4%	3.3%	2.3%	**6.9%**	1.2%	0.6%
Oceanospirillales, SUP05, unknown	**17.2%**	**57.8%**	**65.9%**	0.2%	0.0%	0.0%	0.0%	**21.9%**	**14.6%**	**13.3%**	**25.9%**	**6.0%**	1.0%
Flavobacteriales, Flavobacteriaceae, *Polaribacter*	**16.1%**	**5.5%**	**7.4%**	2.6%	2.6%	**21.3%**	**22.5%**	**22.9%**	**23.6%**	**23.2%**	**21.7%**	**35.5%**	4.5%
Rhodobacterales, Rhodobacteraceae, *Octadecabacter*	**8.0%**	1.1%	1.2%	0.1%	0.0%	**40.0%**	**19.3%**	**5.9%**	**7.6%**	**9.4%**	3.8%	**7.7%**	0.1%
Methylococcales, unknown, unknown	**7.5%**	0.0%	0.1%	0.0%	0.0%	0.0%	0.0%	**15.8%**	**25.4%**	**26.0%**	**22.6%**	0.0%	0.1%
Thaumarchaeota, marine group 1, *Nitrosopumilus*	4.8%	0.2%	0.2%	1.4%	1.6%	**22.3%**	**16.1%**	0.0%	0.0%	0.0%	0.0%	2.4%	**13.0%**
Rhodobacterales, Rhodobacteraceae, unknown	3.3%	0.1%	0.1%	0.1%	0.0%	**10.0%**	**5.2%**	1.0%	1.5%	2.7%	1.2%	**17.3%**	0.8%
Oceanospirillales, Halomonadaceae, *Candidatus portiera*	3.1%	0.0%	0.0%	0.1%	0.1%	0.0%	0.0%	**15.4%**	**9.1%**	**8.1%**	0.8%	0.0%	4.1%
Thiotrichales, Piscirickettsiaceae, unknown	2.8%	0.0%	0.1%	0.0%	0.0%	0.0%	0.1%	4.9%	**8.2%**	**9.0%**	**9.8%**	0.0%	1.7%
Flavobacteriales, Flavobacteriaceae, unknown	1.6%	0.0%	0.1%	0.1%	0.0%	0.0%	0.0%	0.1%	0.1%	0.0%	0.0%	**16.2%**	2.4%

*Values 5% and above in bold*.

Carnivorous sponge samples were dominated by a combination of Proteobacteria and Bacteroidetes, and Thaumarchaeota. The JMVF water sample also included smaller amounts of additional phyla including Verrucomicrobia, Tenericutes, SAR406, Planctomycetes, Cyanobacteria, Chloroflexi, Actinobacteria, and Euryarchaeota. At the class level, Gammaproteobacteria was the most abundant class in *Asbestopluma (Asbestopluma) furcata* and *C. methanophila*. It also completely dominated the *C. abyssicola* and *C. corticocancellata* samples, while other *Cladorhiza* samples were more mixed. Alphaproteobacteria were also present in all samples, and had a large presence in some *Cladorhiza* samples and *L. cupressiformis*. Flavobacteriia were most abundant in *L. cupressiformis*, and were also present in other samples to varying degrees.

Within the Gammaproteobacteria, an unknown genus and family of Thiohalorhabdales was present in all cladorhizid samples. The abundance varied greatly between samples: While the *C. abyssicola* and *C. corticocancellata* samples had over 90% reads from this group (but see caveat regarding sample fixation, section “Run Statistics”), other samples averaged 30% (*C. gelida* specimen 2), ~10–20% (*A. furcata*) or less than 10% for the rest of the samples. The order Oceanospirillales, was prominent in several samples, most significantly in the two *A. furcata* specimens (57.7 and 65.8%), and to a lesser degree in *C. methanophila* (~15–25%) and *L. cupressiformis* (6.0%), but absent in other *Cladorhiza* samples. Most of the Oceanospirillales reads were associated with the SUP05 clade, but two other groups were present in smaller quantities as well. The obligate methanotrophic order Methylococcales (Bowman, 2005) was present in *C. methanophila* only, where it was one of the major groups in terms of abundance (16–25%). Another group found almost exclusively in *C. methanophila* was the order Thiotrichales (5–10%).

The Alphaproteobacteria were mainly represented by Rhodobacterales, especially *Octadecabacter*, which was present in large quantities in *C. gelida* (20–40%), in lesser quantities in *C. methanophila* (3.9–6.0%) and *L. cupressiformis* (7.7%) and almost absent in *A. furcata*, *C. abyssicola*, and *C. corticocancellata* (0–1.2%). Another unknown Rhodobacteraceae was present in slightly lower overall quantities: 17.3% in *L. cupressiformis*, 5–10% in *C. gelid*a and low amounts in other samples (0–1.5%).

The main part of Flavobacteriia reads were identified as *Polaribacter* with 35.6% abundance in *L. cupressiformis*, over 20% abundance in *C. methanophila* and *C. gelida*, and lower values in the range of 2.6–7.4% for *A. furcata*, *C. abyssicola*, and *C. corticocancellata*. A second group of Flavobacteriaceae was also present in the case of *L. cupressiformis* (16.2%), making the total for Flavobacteriaceae over 50% in this specimen. The only archaeal group found in any significant amount was *Nitrosopumilus* from the Thaumarchaeota Marine Group 1, which was found in moderate amounts (16–22%) in *C. gelida* and low amounts (0–2.4%) in other samples.

#### Bray–Curtis and Cluster and NMDS Analyses

**FIGURE 8:**
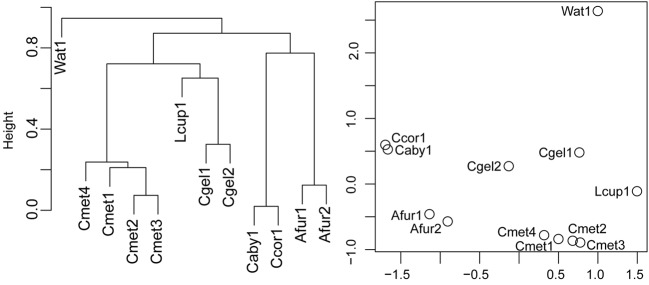
**Cluster analysis and NMDS plot of normalized Bray–Curtis pairwise distances for 97% OTUs from specimens in the dataset**.

Bray–Curtis normalized distances were calculated between specimens on the subsampled microbiome abundance dataset in order to identify systematic differences in microbiome composition. The Bray–Curtis distances were used to perform a hierarchical average linking clustering and NMDS analyses at the 97% OTU level (**Figure 8**). The cluster analysis recovered the water sample in a root position with one clade of *C. methanophila*, *L. cupressiformis*, and *C. gelida* and one clade of *C. abyssicola*, *C. corticocancellata*, and *A. furcata*. However, results show small Bray–Curtis distances (0.7–0.9) between these clades. The NMDS analysis recovered the water sample some distance away from the cladorhizid samples, which broadly cluster in one clade containing *C. gelida* and *C. methanophila*, and one clade containing *C. abyssicola*, *C. corticocancellata*, and *A. furcata*.

## Discussion

### Comparison with Other Sponge Microbiomes

Our results represent the first comparative study of microbiome composition in carnivorous sponges, expanding on the results of the single-species carnivorous sponge study by Dupont et al. (2013) as well as providing a reference for comparison against larger studies of non-carnivorous sponges like Thomas et al. (2016) and deep-sea sponges such as Kennedy et al. (2014).

The class level microbiome community structures observed in this study are similar to previously published filter-feeding sponge microbiomes, with a dominance of Gammaproteobacteria and Alphaproteobacteria (Hentschel et al., 2012; Thomas et al., 2016). Other major groups in our study are Bacteroidetes and Thaumarchaeota, and Bacteroidetes was found to be the dominating group in *L. cupressiformis* (**Figure 6**). Thaumarchaeota are often found as a secondary group in several studies (Taylor et al., 2007; Thomas et al., 2016). Reports of Bacteroidetes are less common, but have been reported for deep-sea sponges (Kennedy et al., 2014), including the previously sequenced microbiome of the carnivorous sponge *L. hypogea* (Dupont et al., 2013).

At higher taxonomic resolution (family and genus level), large variation in the different species microbiomes is evident (**Figure 7**). This is consistent with previous surveys reporting on high degrees of host specificity among sponge microbiomes (Hentschel et al., 2012; Thomas et al., 2016). Moreover, some of the results from sequencing multiple replicates from the same specimen (**Figure 5**) show considerable variation also within each sample, especially for Thiohalorhabdales. While it is possible that the fixative has partly influenced the results in this particular case here (see section “Run Statistics”), the results are in accordance with an uneven distribution of microbial organisms within the host sponges, as previously reported through TEM observations by Riesgo et al. (2007).

Interestingly, an unknown Thiohalorhabdales, and Oceanospirillales, both from the SUP05 clade as well as others, were abundant across all species in the dataset (**Figure 7**; **Table 4**). The only described species of Thiohalorhabdales is *Thiohalorhabdus denitrificans*, an obligate chemolithoautotrophic sulfur-oxidizing bacterium from hyper-saline habitats (Sorokin et al., 2008). Thiohalorhabdales have been reported in low concentrations from sea water with abundance correlated with nitrite concentration (Frank et al., 2016). The Oceanospirillales SUP05 group is also associated with sulfur oxidation, partly at vent and seep sites (Anantharaman et al., 2013). It has been reported both as free-living bacteria and as an endosymbiont (Marshall and Morris, 2013).

We found small to moderate amounts of Thaumarchaeota Marine Group 1 reads belonging to *Nitrosopumilus* in parts of our dataset (**Figure 7**; **Table 4**), mainly in the case of *C. gelida*. This group was also found in moderate quantities in the JMVF water sample. Members of *Nitrosopumilus* are common in seawater and grow by oxidizing ammonium to nitrite (Könneke et al., 2005). Interestingly, sponge symbiont archaeal nitrification has been reported from several studies (e.g., Hoffmann et al., 2009; Liu et al., 2012; Radax et al., 2012), and *Nitrosopumilus* was also found to be the dominating archaeon in part of the specimens in the deep-sea sponges in Kennedy et al. (2014), implying a possible role for this group within carnivorous sponges.

Sulfur- and ammonia-oxidizing symbionts seem to be common in non-carnivorous deep-sea sponges (Nishijima et al., 2010; Arellano et al., 2013), implying that chemolithoautotrophy could be a widespread supplementary source of nutrition for deep-sea sponges in general (Kennedy et al., 2014). Though the taxonomic affinity of the major putative sulfur-oxidizing groups in our dataset (Thiohalorhabdales, Oceanospirillales) were partly different than that of Kennedy et al. (2014) (chiefly Chromatiales, but also some Oceanospirillales and others), this raises the possibility that partial nutrient acquisition from sulfur oxidation could be common in carnivorous sponges as well, though as evidenced by the isotope signatures from our study (**Figure 3**) such activity would be a supplement to carnivory rather than the major mode of nutrition.

Other significant groups present in the dataset were identified as associated with several marine bacterial taxa: For the Alphaproteobacteria, the two major OTUs were somewhat evenly split between an unknown Rhodobacteraceae and the marine genus *Octadecabacter*. The Bacteroidetes were, excepting an unknown Flavobacteriaceae in *L. cupressiformis*, almost exclusively *Polaribacter*, a genus containing several gas vacuolated, marine bacterial species (Gosink et al., 1998) (**Figure 7**; **Table 4**). (For Methylococcales, see section “Trophic Considerations and Extent of Methanotrophy in Carnivorous Sponges”).

### Host Phylogenetic Analysis

The habit of *C. methanophila* is arbuscular, with a great number of filaments extending in all directions from the branches. Together with the types and sizes of its skeletal spicules, the morphology is similar to and suggests a close affinity to a group of *Cladorhiza* species from the North Atlantic. This group includes the three species *C. abyssicola* (type species of the genus), *C. corticocancellata* and *C. gelida*, also examined here, as well as *C. iniquidentata* Lundbeck (1905), *C. oxeata*, and *C. tenuisigma* (Vacelet and Boury-Esnault, 2002; Hestetun et al., in press). The phylogenetic relationships and systematic classification of the carnivorous sponges were investigated in a recent study (Hestetun et al., 2016b), which included most of the North Atlantic cladorhizid species, and sequences from that study were used here with the addition of *C. methanophila* in order to recover its phylogenetic position.

In view of the morphological similarities, the recovery of *C. methanophila* as a close sister taxon to the other arbuscular North Atlantic *Cladorhiza* species in the dataset (**Figure 4**) is not very surprising. *C. methanophila* has also been reported from the 15°20′N fracture zone segment of the Mid-Atlantic Ridge (Hestetun et al., 2015), and has a known depth range of 2600–4900 m. Most other arbuscular *Cladorhiza* species have a somewhat shallower distribution (mainly 200–2000 m), but the closest sister taxon, *C. gelida*, has a eurybathyal range, reported from 500 to 3500 m (Hestetun et al., in press). Given the lack of any evidence of methanotrophic symbiosis in other closely related cladorhizids in the dataset here, a likely hypothesis is that methanotrophy in *C. methanophila* is a rather recent event, possibly occurring as an adaptation to a vent or seep locality either at the BAP or the Mid-Atlantic Ridge.

### Trophic Considerations and Extent of Methanotrophy in Carnivorous Sponges

As deep-sea sponges, cladorhizids are often found in greater concentrations in the enrichment zones surrounding vent and seep systems, where prey is comparatively abundant. However, the large size and great abundance of the shrub-like aggregations of *C. methanophila* at the BAP greatly exceeds normal concentrations of carnivorous sponges, even at other vent sites. Though captured prey items show that *C. methanophila* still retains the ability to acquire nutrients through prey capture (Vacelet and Boury-Esnault, 2002), this suggests that this sponge is able to derive a large amount of nutrients from its chemoautotrophic association (Vacelet et al., 1995; Olu et al., 1997) (compare **Figure 2** for a visual impression).

Results showed that an undescribed Methylococcales OTU was recovered in large abundance from *C. methanophila* specimens, supporting earlier findings describing symbiosis between this species and methanotrophic symbionts. The isotope results, in particular the very light δ^13^C ratios of -60.5 to -66.0‰, are very strong indicators of a symbiotic origin for organic carbon in the host sponge, and consistent with δ^13^C ratios reported in other symbioses from e.g., Gulf of Mexico vent (-51 to -57‰) (Childress et al., 1986) and seep (-74‰) (Cavanaugh et al., 1987) (-45.4 to -39.6‰ and -68.4 to -78.8‰) (MacAvoy et al., 2002) mussels. Surprisingly, they are also lighter than ratios reported for *C. methanophila* from the BAP by Vacelet et al. (1995) (-48.4 to -48.8‰), which could be related to changes in source carbon signature or precise sampling location.

The high sponge concentrations at the BAP, previous TEM and isotope values (e.g., Vacelet et al., 1995), and microbiome and isotope results from our study (**Figure 3**; **Table 2**), clearly show that this species obtains a majority of nutrients from its symbiont relationship rather than through prey capture. The only other sponge where methanotrophic symbiosis has been reported is *Myxilla (Ectyomyxilla) methanophila* Maldonado and Young (1998). However, here the dominating methanotroph is *Methylohalomonas* (Chromatiales, Ectothiorhodospiraceae) (Arellano et al., 2013).

In contrast to *C. methanophila*, close relatives including *C. abyssicola*, *C. corticocancellata*, and *C. gelida*, did not have isotopic ratios suggesting a large contribution from chemolithoautotrophic symbiosis (**Figure 3**) (see Sweetman et al., 2013 for other JMVF biota). Gammaproteobacteria in groups related to known sulfur-oxidizing bacteria were prominent in the microbiome across different cladorhizids in this study. Hydrogen sulfide is present in high concentrations in the high-temperature fluids venting from white smokers at the Troll Wall site at the JMVF (Baumberger, 2011; Dahle et al., 2015). However, sulfur oxidation, which seems to be a common feature in deep-sea sponges, may, if indeed it signifies chemolithoautotrophy, account for a smaller portion of the nutrient uptake than the methanotrophic symbiosis in *C. methanophila*. Thus, chemolithoautotrophic symbiosis as a dominant form of nutrient acquisition within carnivorous sponges seems to be an isolated case rather than a more widespread phenomenon in currently reported species.

## Conclusion

Taken together, the microbiome structures seen in our dataset show a similar dominance of Alpha- and Gammaproteobacteria as that reported for filter-feeding sponges, with some deviation in less dominant groups such as Bacteroidetes and Thaumarchaeota. However, more interestingly, they display marked functional similarities to the results of the filter-feeding (i.e., non-carnivorous) deep-sea sponge species examined by Kennedy et al. (2014) at a higher resolution, sharing ammonia-oxidizing taxa (*Nitrosopumilus*) and both having significant abundances of sulfur-oxidizing bacteria. Thus, it seems that carnivorous sponges overlap with other deep-sea filter-feeding sponges in the functional properties of major groups of their microbiome, though the lack of other comparative data makes it difficult to ascertain whether these taxonomic differences represent a carnivorous sponge-specific microbiome.

The phylogenetic analysis shows that *C. methanophila* is a close relative of other North-Atlantic *Cladorhiza* species. While it is difficult to assess the importance of sulfur and ammonia oxidation in the metabolism of carnivorous sponges in general from the data presented here, the large quantities of methanotrophic symbiont bacteria and low isotopic signature of *C. methanophila* are clear indications that this species relies to a large degree on its chemoautotrophic symbionts, an association that seems to have evolved in this species as a special case rather than in a larger number of related carnivorous sponges.

## Author Contributions

All authors were involved in planning, choosing appropriate methodology, and helped correct and review the manuscript at multiple stages. JH helped collect specimen material, did morphological identification and phylogenetic analysis, conceptualized the article, did part of the 16s rRNA laboratory work, ran 16S rRNA analyses, wrote most of the article, created figures and tables, and implemented changes and corrections to the manuscript. HD helped conceptualize the structure of the article and necessary laboratory work, provided scripts and input on the analytical methodology of the article, and helped plan and perform the 16S rRNA analysis. SJ did part of the 16s rRNA laboratory work, and helped with data analysis. BO helped collect specimen material, prepared samples for isotope analysis, helped interpret isotope values, and helped write parts of the article. HR helped collect specimen material, helped plan the structure of the manuscript, and provided funding for the project.

## Conflict of Interest Statement

The authors declare that the research was conducted in the absence of any commercial or financial relationships that could be construed as a potential conflict of interest.
